# Analysis of the Influence of Hormone Replacement Therapy on Osteocalcin Gene Expression in Postmenopausal Women

**DOI:** 10.1155/2015/416929

**Published:** 2015-08-19

**Authors:** Mansur Rahnama, Izabela Jastrzębska-Jamrogiewicz, Rafał Jamrogiewicz, Grzegorz Trybek

**Affiliations:** ^1^The Chair and Department of Oral Surgery, Medical University of Lublin, Karmelicka 7 Street, 20-081 Lublin, Poland; ^2^The Department of Oral Surgery, Pomeranian Medical University, Al. Powstańców Wlkp. 72, 70-111 Szczecin, Poland

## Abstract

*Background.* Osteocalcin (OC) contributes to the process of bone mineralization. Present study was designed to investigate the changes in OC gene expression of postmenopausal women treated with hormone replacement therapy (HRT). Study was also designed to evaluate OC gene expression in cells which are not part of connective tissue. *Material and Methods.* Research was carried out on 30 postmenopausal women not treated and 30 treated with HRT. Examination of OC gene expression was conducted on peripheral blood lymphocytes (PBL) and buccal epithelial lining (BEL). Densitometry was conducted on femur and mandible. *Results.* Tests revealed OC gene expression in BEL and PBL. BMD was higher in groups treated with HRT. Assessment of correlation between the OC gene expression in BEL and BMD of mandible revealed significant positive relation. *Conclusions.* OC gene expression can be stated BEL and PBL. Analysis of correlation between OC gene expression in oral cavity and mandible BMD showed significant correlation between local OC expression and local bone metabolism. The relation between OC gene expression and bone metabolism is complex and further research is needed to clear all of the uncertainties.

## 1. Introduction

Bone metabolism is a dynamic process which involves bone formation and resorption. Osteoblasts are responsible for bone apposition, while osteoclasts participate in the process of bone resorption. Constant reconstruction of bone tissue is known as remodeling. There are biochemical markers which may be used during the process of monitoring of bone metabolism. Enzymes and proteins released into the circulation during osteoblastic and osteoclastic activity may reflect changes which take place in the skeleton. Changes in concentrations of bone metabolism markers may be used in order to assess the rate of bone turnover [1]. Information about the status of connective tissue is essential for the assessment of the rate of decrease of bone mineral density (BMD), estimation of the risk of bone fracture, and monitoring of connective tissue diseases therapy.

Osteocalcin (OC, bone gamma-carboxyglutamic acid-containing protein (BGLAP)) is a noncollagenous bone matrix polypeptide encoded by the* BGLAP* gene, which is located on chromosome 1 (1q22) [2, 3]. It is produced mainly by osteoblasts. OC is a vitamin K-dependent and vitamin D-dependent protein. Osteocalcin gene expression is controlled by modularly distributed basal and hormone-responsive elements, which are located in DNase I-hypersensitive sites which are present in bone-derived cells [4]. Osteogenesis is mainly controlled by core binding factor alpha 1 (*Cbfa1*), also known as runt-related gene 2 (*Runx2*), which is the transcript factor.* Cbfa1* activates OC gene expression by osteogenic cells [5]. Other important elements which regulate OC gene expression are recognized by the 1*α*,25-dihydroxyvitamin D_3_ receptor (VDR) complex upon ligand activation. This VDR element is located in the distal region of the Osteocalcin promoter and leads to increase in OC gene transcription.

In a carboxylated form, OC binds with calcium and contributes to the process of bone mineralization [6]. Other systemic functions of OC are decrease of visceral fat, increase of energy expenditure, and increase of the number of pancreatic *β*-cells leading to growth of insulin secretion [7]. Increased levels of circulating Osteocalcin can be stated in metabolic diseases connected with accelerated bone turnover, such as the osteoporotic changes in postmenopausal women, particularly in the case of rapid bone loss. Other pathologies which affect the OC concentration include osteomalacia, Paget's disease, thyrotoxicosis, primary hyperparathyroidism, and renal osteodystrophy [8]. Half-life of BGLAP is short and reaches several minutes and its concentration fluctuates considerably during the day (concentration is maximal in the middle of the night and minimal in the morning).

Osteocalcin is mainly produced by mature osteoblasts, but OC expression was also detected in other types of cells. OC immunoexpression was stated in the subcutaneous adipose tissue, used during the treatment of osteolytic bone defects [9]. What is more, the phenomenon of epithelial-to-mesenchymal transition can cause the expression of OC in epithelial tissue. The epithelial-to-mesenchymal transition is crucial, inter alia, for the process of embryonic craniofacial development or wound healing. Transformation of epithelial cells into mesenchymal can lead to recruitment of new osteoblasts or chondrocytes. The process of epithelial-to-mesenchymal transition can be observed during the healing of bone fractures. During this process mesenchymal osteoblasts and chondrocytes which take part in the healing of bony defect stain positive with antibodies which are specific for endothelial markers [10]. Also immune cells are involved in the process of bone healing. Activated immune cells release signaling factors which modulate the function of osteogenic cells. Factors which directly activate lymphocytes are unknown. Expression of major histocompatibility complex (HLA) class II determinants on osteoblasts seems to play an important role in local immune cell activation. HLA positive bone surface cells activate T lymphocytes which affect bone metabolism via multifactorial signaling pathway [11].

The development of postmenopausal osteoporosis is determined by the size of the peak bone mass, which is defined as the highest bone mass achieved during life. Currently, it is believed that 70% of the peak bone mass variation can be attributed to genetic factors. It is suggested that number of specific genes can determine bone mass, its fluctuation, and severity of the resorption process. In addition to environmental and dietary habits affecting skeleton, the bone metabolism is inherited from parents [12]. Polymorphism of several genes encoding the receptors for Osteocalcin is associated with bone density and the risk of fracture [13].

Menopause is defined as termination of menstrual cycles due to the failure of ovarian function (natural menopause—M). Menopausal symptoms also occur after surgical removal of ovaries (ovariectomy—OV)—surgically induced menopause [14]. Cessation of hormonal function of ovaries results in estrogen deficiency, which leads to the development of systemic metabolic disease of bone tissue—postmenopausal osteoporosis. Etiology and pathogenesis of postmenopausal osteoporosis have not been sufficiently explored and still require further investigation.

Hormone replacement therapy (HRT) for a long time has been recognized as a method of treatment in the event to adverse symptoms resulting from the cessation of ovarian endocrine function. HRT is administered in the form of estrogen alone or in the form of estrogen-progestogen therapy. In studies conducted over the past several years, the beneficial prevention of distant effects of menopause, such as osteoporosis or atrophy of skin, caused by HRT has been documented. Estrogen replacement therapy substantially or completely prevents postmenopausal bone loss. If it is used in the early phase of menopause or immediately after ovariectomy, it maintains bone mass. While in women who have started treatment after few years since the last menstrual period, HRT prevents further bone resorption. Estrogen inhibits the release of proinflammatory cytokines and reduces metabolic activity of osteoclasts. It is worth noting that women taking estrogen have fewer fractures than postmenopausal patient who are not treated with HRT [15, 16]. The research work on mechanisms of HRT osteoprotective effect is still carried out in order to potentiate their beneficial effects and minimize complications of estrogen therapy.

The aim of this study was to investigate the expression of Osteocalcin gene in peripheral blood lymphocytes (PBL) and buccal epithelial lining (BEL) of postmenopausal women. Potential differences in the OC gene expression between postmenopausal women treated and not treated with HRT would allow determining if osteoprotective effect of estrogen is exerted, inter alia, by changes in metabolism of OC.

## 2. Materials and Methods

The research was conducted in a group of 30 postmenopausal women undergoing HRT for 6 months (age range 49–59 years; mean age 53.0 years) (study group). Women from study group were supplemented with combination HRT (estrogen and progesterone in combination). Patients received Femoston in tablets (2 mg of estradiol hemihydrate and 10 mg of dydrogesterone). Estrogen was taken on a continuous basis. Patients received progesterone for the last 14 days of each course (course lasted for 4 weeks). The control group consisted of 30 postmenopausal women, at least 36 months after the last menstruation (age range 53–59 years; mean age 55.4 years). Patients in the control group have never received HRT. Patients were treated in the out-patients gynecological clinic of Public Hospital Number 4 in Lublin (Poland). Control and research groups were divided into four subgroups: M: group of postmenopausal women, OV: a group of women after surgical removal of ovaries, OV + HRT: a group of women after surgical removal of ovaries using HR and M + HRT: a group of postmenopausal women treated with HRT. Patients after ovariectomy underwent surgery at least 36 months before the study was conducted. Surgeries were performed as a way of treatment of diseases of reproductive system. In case of patients with malignant tumors, no metastasis had been discovered. None of patients have received neither chemo- nor radiotherapy at least for 2 years before study. Testing procedures received approval of local ethics committee in Lublin (registration symbol KE-0254/246/2005). The study was carried out in accordance with the ethical principles concluded in the Declaration of Helsinki. All patients gave their consent for the examination and research protocol.

Questions included in the anamnesis chart were as follows: age, occupation, socioeconomic status, date of the last menstrual period, duration of hormone replacement therapy (HRT), addictions, physical activity, medications, and surgeries. Women enrolled for the study had no medical history of fractures. None of patients from control group had been treated for osteoporotic changes. None of patients enrolled for tests had been constantly treated with steroids [17]. Patients that qualified for the study did not suffer from any severe systemic diseases, had no addictions, and had not been treated with any medications on constant basis [18]. Patients enrolled for further test were between 36 and 48 months after last menstrual period. Body mass index was calculated for each patient. Patients qualified for the study had BMI values between 27 and 29 (values classified as overweight, but not obese). Examination of the oral cavity was performed with use of dental mirror and periodontal probe. Clinical examination focused on the presence of any pathological mucosal lesions, dental status, presence of dentures, and oral hygiene. Patients that qualified for further tests did not have any acute inflammatory conditions of oral mucosa and the depth of periodontal sockets was less than 5 mm. Patients that qualified for the study did not require any tooth extractions and presented with average or good oral hygiene. Women that qualified for the study did not use any removable dentures. Samples were collected at fixed hours (7–9 a.m.). Epithelial lining of buccal mucosa was collected and suspended in 5 mL of 0.9% saline and centrifuged for 15 minutes at speed of 3000 rpm in order to separate the epithelium. Obtained buccal epithelium and newly drawn, uncentrifuged blood collected in an EDTA tube were used for analysis of gene expression: a control GAPDH gene and Osteocalcin gene [19]. Total cellular RNA isolation was performed using TriReagent Sigma (method modified by Chomczynski and Sacchi) according to the procedure provided by the manufacturer [20]. Following complete dissociation of the nucleoprotein complex (5 minutes at room temperature), 0.2 mL of chloroform was added, shaken vigorously, incubated for 15 minutes, also at room temperature, and centrifuged at a 12,000 rpm for 20 minutes at 4°C. Lysate was divided into three phases: the organic phase containing protein, interphase containing DNA, and colorless upper aqueous phase containing RNA. After transferring the aqueous phase to new Eppendorf test tubes, 0.5 mL of isopropanol was added. The samples were left for 10 minutes at room temperature and then centrifuged at 12,000 rpm for 10 minutes at 4°C. After removing of the supernatant, RNA residue was washed with 75% ethanol, centrifuged (7500 rpm) for 5 minutes, dried, and then dissolved in H_2_O. Obtained RNA was used to prepare cDNA. RNA isolation from blood (1 mL) was preceded by the lysis of erythrocytes in a buffer composed of NH_4_Cl 0.8 M, KHCO_3_ 0.05 M, and EDTA 0.01 M. After 30 minutes of incubation at 4°C, blood was centrifuged (12,000 rpm) for 20 minutes at 4°C. The supernatant was discarded and RNA was isolated from the sediment according to the method described above. cDNA synthesis was performed using a reagent kit: High-Capacity cDNA Reverse Transcription Kit (Applied Biosystems), in accordance with the procedure recommended by the manufacturer. 2 *μ*g of total RNA was used per 20 *μ*L reaction. The 2x RT master mix was prepared using the kit components before preparing the reaction plate. During the procedure Kit with RNase Inhibitor and MultiScribe Reverse Transcriptase was used. 10 *μ*L of 2x RT master mix and 10 *μ*L of RNA sample were pipetted into each well of a 96-well reaction plate or individual tube. The plate was centrifuged to spin down the contents and to eliminate any air bubbles. Reverse transcription was performed in thermal cycler. Step 1 of reaction lasted for 10 min in temperature of 25°C. Second face of process lasted for 120 min in temperature of 37°C. Step 3 of reaction lasted for 5 min in temperature of 85°C. cDNA Reverse Transcription Reactions were stored at temperature of −15 to −25°C. cDNA was used for “real-time” PCR in order to determine the level of GAPDH (control gene) and BGLAP (Osteocalcin gene) expression. TaqMan glyceraldehyde-3-phosphate dehydrogenase Control Reagents [Human] and Bone gamma-carboxyglutamate (gla) protein TaqMan Gene Expression Assay (hCG1999357). The oligonucleotide primer sets which were used for PCR analysis of cDNA were human Osteocalcin 5′-ACACTCCTCGCCCTATTG-3′ (forward) and 5′-GATGTGGTCAGCCAACTC-3′ (reverse) and glyceraldehyde-3-phosphate dehydrogenase (GAPDH) 5′-ACCACAGTCCATGCCATCA-3′ (forward) and 5′-TCCACCACCCTGTTGCTGT-3′ (reverse).

Evaluation of bone mineral density (BMD) of the femur was carried out at the Laboratory of Densitometry at The Institute of Agricultural Medicine in Lublin, by means of the DPX-A (General Electric Healthcare Technologies Lunar) Prodigy Advance with use of Femur option and absorptiometry of X-rays beams of two energies. Scan time lasted for 30 seconds. The BMD was measured at the level of upper femoral neck. The area of interest was determined by analysis software. Images of the mandible were scanned at level of mental foramen and the average bone density of the area was calculated. Bone density was specified in g/cm^2^. In order to evaluate the results of densitometry examination, the* T*-score index was calculated.* T*-score index is a ratio of the patient's bone mineral density and the average bone density of a young person.* T*-score allows stating objectively the loss of bone mass.* T*-score values characterizing the quality of the bone are defined as follows: healthy bone:* T*-score greater than (−1), osteopenia:* T*-score between (−1) and (−2.5), and osteoporosis:* T*-score of less than (−2.5).

Results were statistically analyzed using STATISTICA software. The arithmetic mean (M) and standard deviation (SD) were calculated. The significance of difference between groups was based on confidence intervals determined by the analysis of variance (ANOVA). The interdependence between selected traits was expressed by Pearson's correlation, which is a measure of the dependence between the variables. The Pearson's correlation indicates level of linear dependence between two variables. The Pearson's correlation coefficient between two variables can be defined as covariance of two variables divided by the product of their standard deviations [21]. Value of (+1) stands for a perfect positive (increasing) linear relationship. Value of (−1) stands for a perfect negative (decreasing) correlation. Values approaching (0) show lack of correlation between variables. A paired *t*-test was used to determine whether results differed significantly between control and research groups. The risk of inference error in the study was 5%, which means that the results were significant if *P* value was equal to or less than 0.05.

## 3. Results

Relevant medical information about patients is presented in Table 1. No significant differences between research and control groups were stated in terms of age, time from last menstrual period, duration of hormone replacement therapy, or body mass index (Table 1).

Results of Osteocalcin gene expression in epithelial lining of the cheek revealed slightly higher gene expression in control group, when compared to group treated with HRT. The difference was statistically significant between groups OV + HRT and OV. The highest average OC gene expression was stated in group OV (BGLAP to GAPDH gene ratio = 0.35) and the lowest in group M + HRT (BGLAP to GAPDH gene ratio = 0.03) (Table 2).

Analysis of data concerning the expression of Osteocalcin gene in PBL showed the lowest average level of gene expression in group (OV) (BGLAP to GAPDH gene ratio = 0.67) and the highest in group (OV + HRT) (BGLAP to GAPDH gene ratio = 2.3) and the difference between groups after ovariectomy was statistically significant. BGLAP gene expression was slightly lower in group M + HRT than in group M, but the difference was not statistically significant (Table 2). Comparison of OC gene expression between epithelium of cheek and peripheral blood lymphocytes revealed higher expression in lymphocytes than in epithelial cells (Table 2).

Results of BMD and* T*-score evaluation presented in Table 3 reveal that BMD of femur is significantly higher in groups treated with HRT. Analysis of results for symphyseal area of mandible shows significantly higher level of BMD in group M + HRT when compared to group M. In case of patients after surgical ovariectomy, results show that* T*-score in group treated with HRT is higher than in control group, but the difference is not high enough to be statistically significant.

Assessment of Pearson's correlation between the concentration of OC gene expression in epithelium of the cheek and BMD of mandible revealed significant positive correlation in all groups except in group M. Based on the determined regression line, it can be considered that increase in OC expression resulted in increase of mandible BMD (Table 4). Analysis of the Pearson's correlation for OC gene expression in PBL and BMD of femur showed significant positive relationship only in group M. In group M + HRT linear relationship between Osteocalcin gene expression in the epithelium of the buccal mucosa and in peripheral blood lymphocytes was statistically significant (*r* = 0.8892). This means that, in population of women from group M + HRT, it can be considered that the increase in Osteocalcin gene expression in the epithelium of the buccal mucosa by 0.1 (units) caused an increase in OC gene expression in peripheral blood lymphocytes of 7.07675 (units). Also in the group (OV), correlation between Osteocalcin gene expression in the epithelium and in peripheral blood lymphocytes was high enough to be statistically significant (*r* = 0.6182). This means that, in group (OV), the increase in expression of the Osteocalcin gene in the epithelium by 0.1 (units) caused an increase in Osteocalcin gene expression in peripheral blood lymphocytes of 0.15288 (units). In other groups no significant linear correlation between gene expression in epithelium and blood lymphocytes was stated (Table 4).

## 4. Discussion

Many clinical factors can significantly influence the bone metabolism. Nutrition, general diseases, body mass index, age, or time from last menstrual period substantially affect the processes of remodeling which take place in connective tissue [22, 23]. In order to avoid inaccuracies in tests results, which could be the result of the impact of factors other than the level of sex hormones on bone status, patients that qualified for the study did not differ significantly in terms of age, physical activity, oral hygiene, general health, oral health status, or duration of hormone replacement therapy.

Cessation of ovarian function exerts crucial influence on the organism. One of the adverse effects of estrogen deficiency is the domination of resorption process over apposition of bone tissue [24]. HRT is a therapeutic method used to compensate for bone loss caused by estrogen deficiency in postmenopausal women, but the pathways by which the HTR influences the bone tissue are still not completely known [25]. Influence of Osteocalcin on BMD has been discussed by many authors. The aim of the present study was to investigate the influence of sex hormones supplementation on the expression of BGLAP gene.

Osteoporosis is a disease of bone tissue, which leads to an increased risk of fracture. It leads to BMD reduction and bone microarchitecture alteration. Osteoporosis starts with decalcification—a condition called osteopenia. Many previous studies support the theory of an inverse correlation between serum biomarker concentration and the level of estrogen expressed by increased level of serum Osteocalcin. Study conducted by Laloš-Miljuš et al. revealed a difference between concentrations of OC in patients suffering from osteoporosis or osteopenia [26]. Levels of OC in patients with osteopenia were significantly lower (29.26 ± 3.65 ng/mL) than in patients diagnosed with osteoporosis (32.07 ± 6.24 ng/mL). Fluctuations in OC levels in the course of postmenopausal osteoporosis were presented in the study carried out by Gurban and associates [18]. Levels of OC among women who had not been menstruating for at least 15 years reached values of 20.12 ± 0.87 ng/mL, whereas in group where this period was less than 15 years concentrations of OC were significantly lower (15.12 ± 1.55 ng/mL). These results allow for putting forward a thesis that sustained decrease in function of osteoblasts after the last menstrual period is reflected by increased levels of ucOC in blood serum. This assumption may be relevant in the understanding of the OC metabolism. Lower female sex hormone levels cause changes in the OC system, leading to the decrease of OC and in consequence increase in ucOC levels in blood serum. However, not all authors completely agree with this theory. A study carried out by Lewandowski et al. showed that the level of secretion and concentration of Osteocalcin in body fluids was influenced by various factors, such as medicines, general diseases, or diet [27].

Correlation between Osteocalcin gene expression and bone metabolism was previously investigated by Rodrigues et al. [28]. In their study conducted on 64 patients (25 subjected to hip replacement surgery due to pathological fractures and 39 due to osteoarthritis), authors compared the ratios of bone OC/collagen gene expression (OC/COL1A1). The ratio was significantly lower among patients with hip fracture compared to those with osteoarthritis. Low bone OC/COL1A1 expression ratio was a predictor of worse trabecular mechanics and of a hip fracture episode. However, analysis of total OC and ucOC in the serum did not show significant differences between groups. Tests performed in present study proved that expression of OC gene is present both in buccal mucosa and in peripheral blood lymphocytes. The expression of Osteocalcin gene in cells which are not directly connected with bone metabolism is probably associated with constant bone remodeling processes which take place in living organism. Immune cells, which are important part of processes of skeleton destruction and healing, and cells from BEL, which overcome epithelial-to-mesenchymal transition, play significant role in metabolism of connective tissue [9–11]. This finding seems to be important due to the fact that bony material for diagnostic tests is far more difficult to obtain than blood or mucosal lining. What is more, the surgical procedure which is needed in order to obtain bone tissue is more complicated and associated with far more severe complications than collecting blood or epithelium samples. Analysis of BGLAP gene expression in present study revealed a statistically significant difference between the OV + HRT and OV groups. Expression of Osteocalcin in BEL was significantly lower in the OV + HRT group when compared to the OV group. An opposite correlation was observed in case of OC gene expression in PBL. Analysis of Pearson's correlation of BGLAP gene expression in the epithelial lining and PBL revealed a statistically significant positive interdependence in OV and M + HRT groups (*r* > 0.5). What is interesting is that the analysis of Pearson's correlation showed that in terms of OC gene expression in cheek epithelium lining in most of groups the gene expression caused increase in the BMD of mandible. Results for PBL did not reveal similar correlations.

It needs to be emphasized that the local levels of biomarkers, such as OC concentration in saliva, can be significantly influenced by factors such as periodontal status or local inflammations and they must be taken into consideration while qualifying patients for tests. In order to avoid significant impact of local inflammatory conditions on BEL OC gene expression, the present study included patients without any foci of acute inflammation in oral mucosa and the depths of their periodontal sockets were less than 5 mm. Patients enrolled for this study did not require any tooth extractions and presented with average or good level of oral hygiene. Another factor which has significant impact on bone condition is body fat content. Increased aromatization of androgens into estrogens takes place in adipose tissue. Adipose tissue functions as an active endocrine organ. Fat secretes hormones and cytokines such as resistin, leptin, adiponectin, TNF, and IL-6, which exert significant influence on the entire human organism, including bone tissue [29]. Increased aromatization of androgens into estrogens in fat tissue leads to elevation of concentrations of endogenous estrogens. Adipose tissue has no direct effect on the production of Osteocalcin, but it affects its concentration in body fluids through substances such as leptin or secreted cytokines [30]. In the present study, patients from both the control and the study group were matched in terms of BMI. Although all patients in our study had a body mass index higher than 25 (above normal), but none of the patients were categorized as obese (BMI lower than 30). Patients were matched in terms of BMI in order to prevent significant influence of adipose tissue on OC gene expression.

Osteocalcin gene expression can be influenced by many factors such as diet, steroid hormones, or inflammatory cytokines. Moreover, levels of biomarkers significantly changed throughout the day [31]. In their study, Gouveia et al. demonstrated the influence of thyroid hormones on the expression of OC [32]. OC secretion is influenced not only by estrogen but also by other hormones, such as thyroid hormones, growth hormone, or glucocorticoids [32, 33]. Patients included in the study did not receive any medications or dietary supplementation. Samples were collected at the same hours in order to avoid fluctuations in gene expression. It is impossible to eliminate all factors influencing OC level fluctuations. Moreover, levels of bone turnover biomarkers such as Osteocalcin are not specific for diseases of bone metabolism. Fluctuations in the expression of BGLAP indicate changes in bone metabolism regardless of their cause. Increased levels of OC may appear not only in osteoporosis but also in other diseases of bone tissue, such as hyperparathyroidism, Paget's disease, bone neoplasm, or avitaminosis. A study by Lee et al. showed that the level of OC may be significantly influenced by metabolic disorders and could be a predictor of abdominal obesity or insulin resistance [34]. Ducy reported that Osteocalcin-null mice exhibit increased apposition of bone tissue without impaired osteolysis. This finding may suggest that interaction between recruited osteoblasts and osteoclast in the process of bone remodeling is complex and not completely dependent on the OC expression [5]. These factors greatly limit use of OC as a reliable indicator of osteoporosis in menopausal women. The analysis of correlation of OC gene expression and BMD in present study clearly indicates that it is difficult to state a significant correlation between status of skeleton mineralization and OC gene expression in PBL. Results from limited, local area, which is oral cavity, showed more significant relationship between OC gene expression and BMD of mandible. It needs to be stressed out that the OC gene expression was slightly lower in groups treated with HRT than in control groups. Further investigations on a broader group of patients are needed to verify process influencing the metabolism of OC in menopausal women.

## 5. Conclusions

1. OC gene expression can be stated in both peripheral blood lymphocytes and epithelial lining of cheek.
2. Hormone replacement therapy does not significantly influence OC gene expression in PBL/epithelial lining of women during natural menopause.
3. HRT has significant influence on OC gene expression in PBL/epithelial lining of women during surgically induced menopause.
4. The level of OC gene expression in blood stream is highly variable and seems to be influenced by many systemic factors, which makes it difficult to use for diagnostic purposes. Analysis of correlation between OC gene expression in inflammation-free area of oral cavity and BMD of mandible allowed stating of significant relationship between OC and bone metabolism.
5. The relation between OC expression and bone metabolism is complex and further research is needed to clear all of the uncertainties.

## Figures and Tables

**Table 1:** Information about patients enrolled for the study.

Research group	Sample number	Age [years] (M ± SD)	Control group	Sample number	Age [years] (M ± SD)	Statistically significant differences between results in control and research group (*P* < 0.05)
M + HRT	15	53,27 ± 3,13	M	15	55,20 ± 2,21	No
OV + HRT	15	52,73 ± 2,81	OV	15	55,60 ± 2,10	No

		Time from last menstrual period [months] (M ± SD)			Time from last menstrual period [months] (M ± SD)	

M + HRT	15	43,3 ± 3,8	M	15	41,5 ± 3,9	No
OV + HRT	15	41,5 ± 5,1	OV	15	42,7 ± 4,1	No

		Duration of HRT [months] (M ± SD)			Duration of HRT [months] (M ± SD)	

M + HRT	15	17,0 ± 3,8	M	15	17,6 ± 3,6	No
OV + HRT	15	17,6 ± 4,1	OV	15	16,8 ± 3,5	No

		Body mass index (M ± SD)			Body mass index (M ± SD)	

M + HRT	15	27,73 ± 0,70	M	15	27,87 ± 0,83	No
OV + HRT	15	27,87 ± 0,83	OV	15	27,80 ± 0,77	No

**Table 2:** Ratio of Osteocalcin to control GAPDH gene relative expression in epithelial lining of the cheek and peripheral blood lymphocytes.

Research group	Sample number	Ratios of Osteocalcin to control GAPDH gene expression level in the epithelial lining of the cheek (M ± SD)	Control group	Sample number	Ratios of Osteocalcin to control GAPDH gene expression level in the epithelial lining of the cheek (M ± SD)	Statistically significant differences between results in control and research group (*P* < 0.05)
M + HRT	15	0,03 ± 0,02	M	15	0,11 ± 0,17	No
OV + HRT	15	0,05 ± 0,05	OV	15	0,35 ± 0,21	Yes

		Ratios of Osteocalcin to control GAPDH gene expression level in peripheral blood lymphocytes (M ± SD)			Ratios of Osteocalcin to control GAPDH gene expression level in peripheral blood lymphocytes (M ± SD)	

M + HRT	15	1,57 ± 1,22	M	15	2,02 ± 2,08	No
OV + HRT	15	2,3 ± 1,72	OV	15	0,67 ± 0,51	Yes

**Table 3:** *T*-score of mandible and femoral bone.

Research group	Sample number	*T*-score of femoral bone (M ± SD)	Control group	Sample number	*T*-score of femoral bone (M ± SD)	Statistically significant differences between results in control and research group (*P* < 0.05)
M + HRT	15	−0,82 ± 0,35	M	15	−1,89 ± 0,87	Yes
OV + HRT	15	−0,90 ± 0,38	OV	15	−1,66 ± 0,49	Yes

		*T*-score of the mandible symphyseal area (M ± SD)			*T*-score of the mandible symphyseal area (M ± SD)	

M + HRT	15	−0,24 ± 0,38	M	15	−0,80 ± 1,41	Yes
OV + HRT	15	−0,63 ± 0,93	OV	15	−1,07 ± 0,74	No

**Table 4:** Correlation between Osteocalcin to control GAPDH gene expression level and BMD.

Group	Sample quantity	Pearson's correlation between ratio of Osteocalcin to control GAPDH gene expression level in the epithelial lining of the cheek and BMD of mandible—area of teeth 35–45	Pearson's correlation between ratio of Osteocalcin to control GAPDH gene expression level in peripheral blood lymphocytes and BMD of femoral bone	Pearson's correlation between ratio of Osteocalcin to control GAPDH gene expression level in peripheral blood lymphocytes and epithelial lining of the cheek
M	15	−0,3367	0,5797	0,1125
OV	15	0,9846	−0,2088	0,6182
M + HRT	15	0,6877	0,2844	0,8892
OV + HRT	15	0,7774	−0,3063	−0,0688
